# Mulberry-extract improves glucose tolerance and decreases insulin concentrations in normoglycaemic adults: Results of a randomised double-blind placebo-controlled study

**DOI:** 10.1371/journal.pone.0172239

**Published:** 2017-02-22

**Authors:** Mark Lown, Richard Fuller, Helen Lightowler, Ann Fraser, Andrew Gallagher, Beth Stuart, Christopher Byrne, George Lewith

**Affiliations:** 1 Primary Care & Population Sciences, Faculty of Medicine, University of Southampton, Aldermoor Health Centre, Southampton, United Kingdom; 2 Functional Food Centre, Oxford Brooks University, Gipsy Lane Campus, Oxford, United Kingdom; 3 Chief Operating Officer, Phynova Group Ltd, 16 Fenlock Court, Long Hanborough, United Kingdom; 4 Nutrition and Metabolism, Faculty of Medicine, University of Southampton and University Hospitals Southampton, United Kingdom; 5 Southampton National Institute for Health Research, Biomedical Research Centre, University Hospital Southampton, Southampton, United Kingdom; Weill Cornell Medical College Qatar, QATAR

## Abstract

**Background:**

High sugar and refined carbohydrate intake is associated with weight gain, increased incidence of diabetes and is linked with increased cardiovascular mortality. Reducing the health impact of poor quality carbohydrate intake is a public health priority. Reducose, a proprietary mulberry leaf extract (ME), may reduce blood glucose responses following dietary carbohydrate intake by reducing absorption of glucose from the gut.

**Methods:**

A double-blind, randomised, repeat measure, phase 2 crossover design was used to study the glycaemic and insulinaemic response to one reference product and three test products at the Functional Food Centre, Oxford Brooks University, UK. Participants; 37 adults aged 19–59 years with a BMI ≥ 20kg/m^2^ and ≤ 30kg/m^2^. The objective was to determine the effect of three doses of mulberry-extract (Reducose) versus placebo on blood glucose and insulin responses when co-administered with 50g maltodextrin in normoglycaemic healthy adults. We also report the gastrointestinal tolerability of the mulberry extract.

**Results:**

Thirty-seven participants completed the study: The difference in the positive Incremental Area Under the Curve (pIAUC) (glucose (mmol / L x h)) for half, normal and double dose ME compared with placebo was -6.1% (-18.2%, 5.9%; p = 0.316), -14.0% (-26.0%, -2.0%; p = 0.022) and -22.0% (-33.9%, -10.0%; p<0.001) respectively. The difference in the pIAUC (insulin (mIU / L x h)) for half, normal and double dose ME compared with placebo was -9.7% (-25.8%, 6.3%; p = 0.234), -23.8% (-39.9%, -7.8%; p = 0.004) and -24.7% (-40.8%, -8.6%; p = 0.003) respectively. There were no statistically significant differences between any of the 4 groups in the odds of experiencing one or more gastrointestinal symptoms (nausea, abdominal cramping, distension or flatulence).

**Conclusions:**

Mulberry leaf extract significantly reduces total blood glucose rise after ingestion of maltodextrin over 120 minutes. The pattern of effect demonstrates a classical dose response curve with significant effects over placebo. Importantly, total insulin rises were also significantly suppressed over the same time-period. There were no statistically significant differences between any of the treatment groups (including placebo) in the odds of experiencing one or more gastrointestinal symptoms. Mulberry extract may have multiple modes of action and further studies are necessary to evaluate ME as a potential target for the prevention of type 2 diabetes and the regulation of dysglycaemia.

## Introduction

Excess calorie intake including those from sugar and carbohydrates along with inactivity can make a significant contribution to becoming overweight [1,2] and thus increase the risk of developing Type 2 diabetes mellitus (T2DM) [3, 4]. In 2013 a large long-term European study investigating the effect of diet on health [5] found an association between the amount of sugary soft drinks people consumed and their risk of T2DM. In the study, weight gain had a large effect on diabetes risk and sugary drinks had a small effect on diabetes risk even after Body Mass Index (BMI) was corrected for [5]. The global rise in T2DM is linked to the metabolic syndrome (dyslipidemia, hypertension, insulin resistance), and obesity is thought to be one of the greatest risk factors for metabolic syndrome and T2DM [6]. Dietary sugars and carbohydrates play a significant role as calories from these foods promote fat storage and hunger [7]. A recently completed review of nutrition and its impact on T2DM concluded that dietary restriction of carbohydrate intake is the single most effective approach to manage T2DM [8]. It is estimated that more than 1 in 17 people in the UK have diabetes (diagnosed or undiagnosed) [9] and thus reducing the health impact of poor quality carbohydrate intake is a public health priority. Herbal agents could be effective in reducing post-prandial blood glucose in combination with carbohydrate restriction [10]. Indeed, the history of the widely prescribed agent Metformin (dimethylbiguanide) can be traced back to the use of *Galega officinalis* Linn as a herbal medicine in medieval Europe [11].

Mulberry (*Morus alba*) leaves have been used in traditional Chinese medicine (TCM) for several millennia and its use was first recorded in around 500AD in the *Divine Husbandman’s Classic of the Materia Medica* [12]. In the Grand Materia Medica, it states "if the juice (of the herb) is decocted and used as a tea substitute it can stop wasting and thirsting disorder.” Reports have shown that the leaves are nutritious and non-toxic [13]. The Chinese Ministry of Health and the Taiwanese Bureau of Food Safety recognise *Morus alba* leaves as both a food and a medicine [14]. Mulberry leaf extracts (ME) have a history of safe ‘traditional’ use for normalizing post-prandial blood glucose, and it is thought that iminosugars such as 1-deoxynojirimycin (DNJ), a reversible, competitive natural *α*-glucosidase inhibitor, are the main active components responsible for the activities [10]. ME 1000-fold diluted has also been shown to inhibit absorption of sucrase, maltase, isomaltase, trehalase and lactase (by 96%, 95%, 99%, 44% and 38% respectively) [10]. ME also contains gallic acid and may have additional anti-diabetic effects via translocation of the GLUT4 receptor [15]. As ME inhibits the absorption of carbohydrates from the intestine, GI side effects are possible.

Previous research has suggested that ME could significantly reduce the peak blood glucose levels and insulin response levels [16,17], providing protection to blood glucose metabolic function of healthy and hyperglycemic subjects [18]. Long-term administration of ME produced a dose-dependent decrease in body weight and hepatic lipid accumulation [19], stimulated skeletal muscle 5'-AMP-activated protein kinase activity acutely without changing the intracellular energy status [20], suppressed the elevation of postprandial blood glucose and cholesterol in humans [16] and exhibited potential hypoglycemic and hypolipidemic effects in patients with diabetes [21]. ME has been shown to suppress postprandial glucose and insulin in healthy human subjects when added to confections in a small study with ten healthy females [22]. Sucrose and starch absorption was inhibited and they were subsequently fermented by intestinal microbiota which could lead to an additional beneficial prebiotic effect [22].

While Mulberry tea has been shown to suppress the postprandial rise of blood glucose levels after 90 minutes of its consumption in T2DM subjects [23] the interpretation of the clinical relevance of the effects of ME has been challenging due to limitations including study design and small numbers of subjects [10,16,17,21–23]. High quality, double blind placebo controlled trials are therefore required to determine the effects of ME on glucose tolerance and to ascertain its potential as a target for further investigation for the prevention of T2DM and regulation of dysglycaemia. We aimed to investigate the effects of ME in healthy volunteers with a high quality placebo controlled clinical trial in the UK.

## Materials and methods

### Study design

The primary outcome of the study was to test the effect of three doses of mulberry-extract (250mg Reducose containing 12.5mg DNJ), half (125mg Reducose containing 6.75mg DNJ) and double (500mg Reducose containing 25mg DNJ) the normal dose of a proprietary water extract of mulberry leaves standardized to contain 5% DNJ (Reducose), versus placebo, on blood glucose (pIAUC for glucose concentration over 120 minutes) when co-administered with 50g maltodextrin in normoglycaemic healthy adults. Secondary outcomes were to test the insulin response (pIAUC for insulin concentration over 120 minutes) and gastrointestinal tolerability of the mulberry extract using normal, half and double the normal dose of ME and placebo. Maltodextrin is a dietary starch with a high glycaemic index and is commonly added to many foods and beverages. The exact dosage regime investigated was determined by a series of initial phase 1 studies carried out on normal healthy subjects by Phynova, the company that owns and produces Reducose. A double-blind, randomised, repeat measure, crossover design trial was used to study the glycaemic response (GR) and insulinaemic response (IR) to three products: one reference product and three test products. Participants acted as their own controls. The trial was conducted at the Functional Food Centre at Oxford Brookes University. The Centre is internationally renowned for its work on GR with extensive publications and their procedure for glycaemic index testing is based on well-established FAO/WHO guidelines. Ethical approval for the study was obtained from the Oxford Brookes University Research Ethics Committee (UREC Registration No: 140806 for glycaemic response (2014); UREC Registration No: 110594 for insulaemic response (2012)). The exclusion criteria of the MULBERRY trial are listed in Table 1. The Study design, rationale and methodology have been previously described in detail [24].

**Table 1:** Exclusion criteria of the mulberry trial.

Exclusion Criteria
Aged < 18 or > 60 years Pregnant or lactating Body mass index (BMI) < 20kg/m^2^ and > 30kg/m^2^ Fasting blood glucose value > 6.1 mmol/l Any known food allergy or intolerance including mulberry extract Medical condition(s) or medication(s) known to affect glucose regulation or appetite and/or influence digestion and absorption of nutrients Known history of diabetes mellitus (Type I/II) or the use of antihyperglycaemic drugs or insulin to treat diabetes and related conditions Use steroids, protease inhibitors or antipsychotics (all of which have major effects on glucose metabolism and body fat distribution) Current oral hypoglycaemic use Symptomatic IBS History of renal or liver diseases History of clotting or bleeding disorders Taken antibiotics in last 3 weeks prior to screening Taking daily medications or dietary supplements that are not suitable for the study in the opinion of the PI Anaemia Subject to a major medical or surgical event requiring hospitalization within the preceding 3 months Current participation in another clinical study.

### Trial registration

ISRCTN: ISRCTN 14597438

### Recruitment

Participants were recruited following local advertisements. All participants were given full details of the study protocol and the opportunity to ask questions. They subsequently gave written informed consent prior to participation and were paid £10 per visit, on completion of all four visits. This was determined as an appropriate amount to cover travel costs and the time spent during each visit. The trial was registered on 21/04/2015 and the first patient recruited on 22/04/2015. The last patient was followed up and the study completed on 29/08/2015. The authors confirm that all ongoing and related trials for this intervention are registered.

### Mulberry leaf extract

Reducose is a mulberry leaf extract standardised to contain 5% (+/- 10%, i.e. 4.5%-5.5%) 1-deoxynojirimycin (DNJ). Batch-to-batch consistency is maintained through a quality control (QC) process that starts with the raw material to ensure the leaves contain a minimum required DNJ content. Production yields batches with >5% DNJ and the content is standardised through batch blending and dilution with excipients. All batches are subjected to rigorous QC during manufacturing and each batch is quantitatively (HPLC-ELSD) assayed for DNJ and qualitatively fingerprinted using HPTLC. All batches undergo routine quality control to ensure contaminant levels (heavy metals, microbes) are within the European pharmacopoeia limit. The exact dosage regime investigated was determined by a series of initial phase 1 studies carried out on normal healthy subjects by Phynova, the company that owns and produces Reducose.

### Randomisation

Participants and investigators were blinded. Participants were assigned a participant number according to their chronological order of enrolment in the study. The allocated participant number was used to identify the participants and their corresponding intervention sequence. Four products were tested in this study—one placebo reference product (four capsules containing 125mg microcrystalline cellulose) and three test products containing different doses of mulberry extract (test product groups received either 1, 2, or 4 capsules containing 125mg ME, with either 3, 2, or 0 placebo capsules respectively so that participants always took 4 capsules). Each test/reference product was co-administered with 50g maltodextrin dissolved in 250ml water.

The reference product and test products were administered to participants in a randomised, repeated measures design. All volunteers received the reference product and test products in random order on (four) separate days, with at least a two-day gap between measurements to minimise carry over effects. DNJ has a relatively short half-life in vivo of approximately 2 hours (when measured in rats using hydrophilic interaction chromatography coupled to a mass spectrometric detector [25]).

### Study procedures

On the day prior to a test, participants were asked to restrict their intake of alcohol and caffeine-containing drinks and to restrict their participation in intense physical activity (for example, long periods at the gym, excessive swimming, running, aerobics). Participants were also told not to eat or drink after 10.00 pm the night before a test, although water was allowed in moderation. Participants were studied in the morning after an overnight fast. Anthropometric measurements (height, weight and BMI) were taken before any products were consumed. Body composition measurements (Fat Mass (FM), Fat-Free Mass (FFM)) were taken using the Tanita BC-418MA segmental body composition analyser. Participants consumed the products at a comfortable pace, within 5 minutes and the reference product and test products were served with 50g maltodextrin dissolved in 250 ml water.

Participants remained sedentary during each test session and did not consume any additional food or fluid. They were instructed to record stool consistency for the first bowel movement after their visit and the frequency and intensity of gastro intestinal symptoms for 0–24 hours after the study product consumption. Gastrointestinal symptoms were measured via questionnaire for 24 hours following each study visit. Subjects used a 5-point scale to rate stool consistency for each bowel movement for 0–24 h after the study product consumption. The five-point scale includes: 1 = watery, 2 = loose/mushy, 3 = soft, 4 = formed, 5 = hard. Frequency and intensity were recorded using a 10-centimeter (cm) line scale (0 representing “Absent” for frequency and “Usual” for intensity; 10 representing “More than usual” for frequency and “Severe” for intensity).

### Laboratory measurements

The glycaemic response method used was adapted from that described by Brouns *et al* [26] and was carried out in accordance with the ISO 26642:2010 standards. Blood measurements were taken at -5 min and 0 min before consumption of the reference product/test products and the baseline value taken as a mean of these two values. Further blood measurements were taken at 15, 30, 45, 60, 90 and 120 minutes after the start. Blood glucose was measured using the HemoCue Glucose 201+ analyser (HemoCue Ltd). The same time points were used for determining insulin levels. At each test time point, 300 μL of capillary blood (from finger pricks) was obtained using the Unistik 3 single-use lancing device (Owen Mumford, Woodstock, UK) and collected into chilled Microvette capillary blood collection tubes treated with di Potassium EDTA (CB 300 K2E; Sarstedt Ltd., Leicester, UK). The Microvette tubes were centrifuged and 200 μL of the supernatant plasma obtained. Insulin concentrations in the plasma samples were determined by electrochemiluminescence immunoassay using an automated analyzer (Cobas E411; Roche diagnostics, Burgess Hill, UK). The Cobas system is a reliable method of plasma insulin determination. Sufficient blood was taken to enable a second set of analysis to be performed at every time point (if the first analysis failed) and there was no missing data. The second sample was used for two participants due to faulty equipment but only one data value at each time point was obtained in all subjects.

### Sample size

A recent unpublished phase 1 study in 12 healthy individuals age 18–25 using 250mg ME dose showed a reduction in the glycaemic index of maltodextrin by 58% when compared to placebo. We estimated a sample size of n = 30 participants would provide over 90% power to detect a similar size of effect. Being more conservative and allowing for a smaller difference to be detected in the lower concentration doses, 30 participants would still allow at least 80% power to detect a difference of 25% in the positive Incremental Area Under the Curve (pIAUC). In order to account for a potential loss to follow up, and the possibility that our sample size may be inaccurate as it is based on a small pilot sample we aimed to recruit 40 participants.

### Statistical analyses

We calculated the positive incremental area under the curve for the 4 study products and compared using repeated measures ANOVA to determine whether there was a statistically significant difference in the primary outcome (glucose response over 120 minutes) and in the secondary outcome measures (insulin response over 120 minutes and gastrointestinal side effects). Repeated measures ANOVA were used to compare treatments across time-points, recognising that responses were clustered within individual participants. For binary outcomes, results are expressed as proportions and repeated measures logistic regression was used (Stata’s xtlogit command). All analyses were carried out in Stata v12.1. The presence/absence of gastrointestinal symptoms in the 24 hours following the study visit was assessed using logistic regression models.

## Results

**Fig 1:**
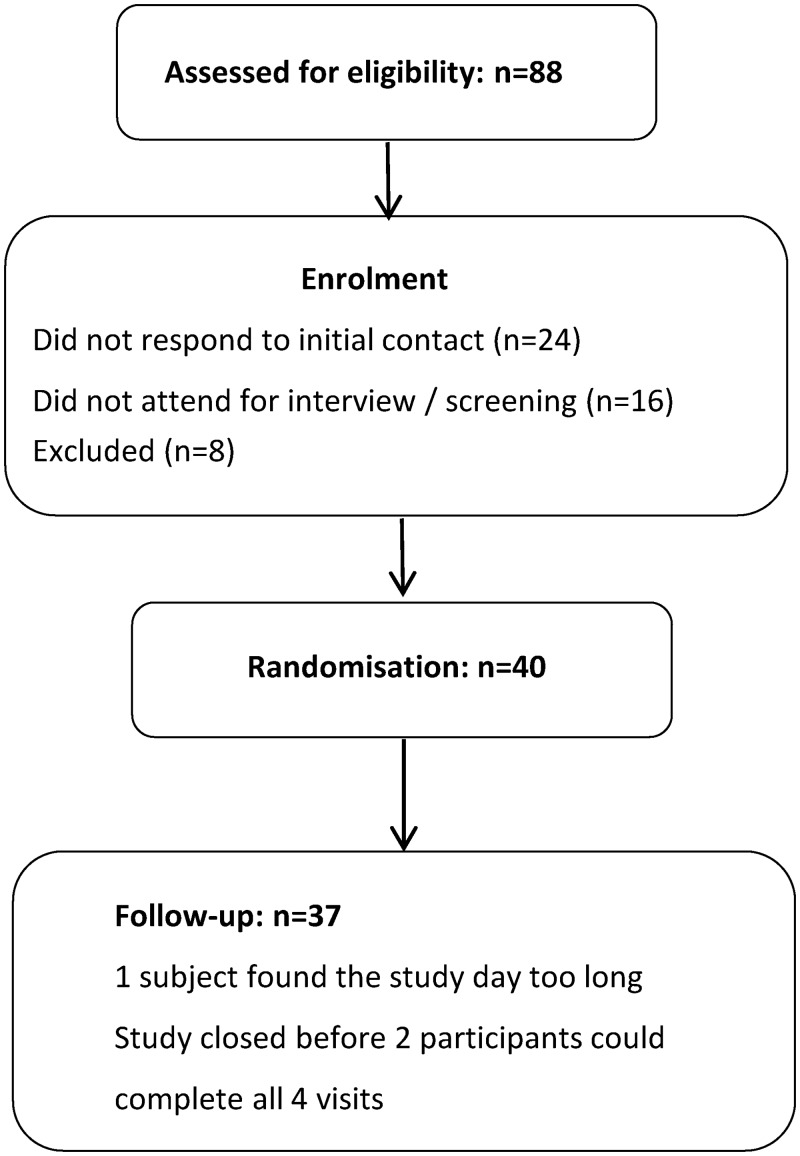
Mulberry study CONSORT diagram.

Of 40 randomised subjects, three participants dropped out (one found the study day too long, and the study was closed before two other participants could complete the remaining visits). Recruitment began in April 2015 and the study was closed at the end of August 2015 with 37 participants having completed all four visits. Fig 1 depicts the trial flow diagram.

37 participants completed the study and the baseline characteristics are shown in Table 2. Positive incremental area under the curve was calculated for all glucose and insulin measurements from baseline to 120 minutes in accordance with FAO/WHO’s ‘*Joint Guidelines on glycaemic index testing of foods*’ and the International Standard *‘ISO 26642/2010*: *Food Products–determination of the glycaemic index (GI) and recommendation for food classification*’.

**Table 2:** Baseline characteristics of the study population.

Characteristic	Male	Female	Total sample
**Female**			25/37 (67.6%)
**Age**	27.17 (7.51)	30.40 (12.24)	29.35 (10.93)
**Height (cm)**	173.08 (6.49)	164.40 (6.28)	167.22 (7.49)
**Weight (kg)**	70.74 (7.35)	61.37 (6.98)	64.41 (8.29)
**BMI**	23.61 (2.09)	22.71 (2.34)	23.00 (2.27)
**Waist circumference (cm)**	81.72 (4.99)	76.46 (6.52)	78.17 (6.50)
**Hip circumference (cm)**	99.30 (4.00)	99.20 (6.65)	99.24 (5.86)
**FM(%)**	15.02 (4.44)	28.94 (5.46)	24.43 (8.34)
**FM (kg)**	10.65 (3.53)	18.06 (5.19)	15.65 (5.84)
**FFM(%)**	84.98 (4.44)	71.06 (5.46)	75.57 (8.34)
**FFM(kg)**	60.09 (6.91)	43.31 (3.18)	48.75 (9.21)

Unless otherwise stated, data are means (SD), (FM–Fat Mass, FFM–Fat-Free Mass).

### Positive incremental area under the curve–Glucose

**Fig 2:**
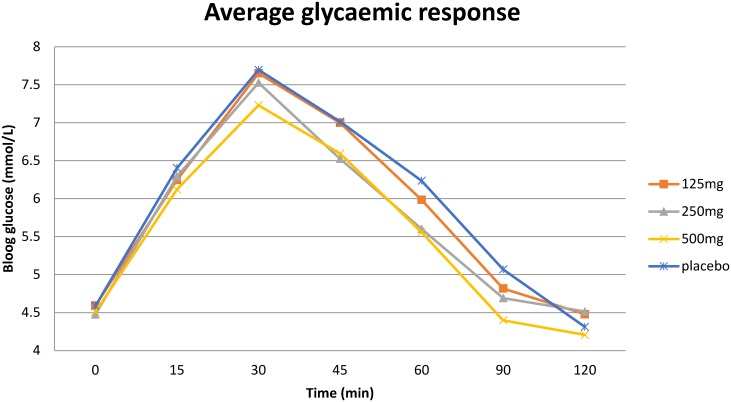
Mean plasma glucose concentrations according to group during the maltodextrin tolerance test.

As shown in Table 3, there are significant differences in the positive incremental area under the curve between treatments. Compared to the placebo dose, the positive incremental area under the curve was significantly lower in the 250mg and 500mg doses. The pIAUC for the 125mg dose was not significantly different from placebo. The 500mg dose also had an area under the curve 0.44 mmol / L x h (95% CI -0.78, -0.11) lower than the 125mg dose. This was statistically significant (p = 0.010). None of the other pairwise comparisons were statistically significant. The average glycaemic response for the four groups is shown in Fig 2.

**Table 3:** Positive incremental area under the curve for glucose.

	Positive incremental area under the curve (mmol / L x h)	Difference compared to placebo (mmol / L x h)
Placebo	2.81 (1.19)	
125 mg	2.64 (1.35)	-0.17 (-0.51, 0.16; p = 0.316)
250 mg	2.42 (1.27)	-0.393 (-0.73, -0.06; p = 0.022)
500 mg	2.19 (0.99)	-0.62 (-0.95, -0.01; p<0.001)

Difference compared to placebo calculated using repeated measures ANOVA model

### Subgroups

Two planned subgroup analyses were to be carried out. Although not powered to detect statistically significant differences within subgroups, exploratory analysis could help to determine whether there is any signal to support hypotheses that differential effects would be observed in those aged over 50 years and in those with a BMI greater than 25 kg/m^2^. There were only two individuals aged > 50 years and therefore this subgroup analysis was not carried out. Similarly, there were no participants with a BMI > 25 kg/m^2^.

### Positive incremental area under the curve–Insulin

**Fig 3:**
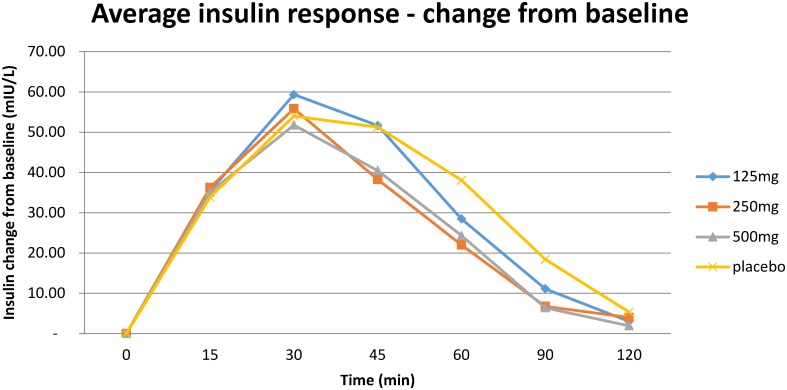
Mean plasma insulin concentration according to group during the maltodextrin tolerance test.

As shown in Table 4, the placebo group had significantly higher pIAUC than the 250mg or 500mg treatments. There were no other statistically significant differences at the 5% level. Fig 3 shows the average insulin response of the groups.

**Table 4:** Positive incremental area under the curve for insulin.

	Positive incremental area under the curve (mIU / L x h)	Difference compared to placebo (mIU / L x h)
Placebo	59.9 (48.5)	
125mg	54.1 (34.5)	-5.83 (-15.5, 3.8; p = 0.234)
250mg	45.6 (22.9)	-14.3 (-23.9, -4.6; p = 0.004)
500mg	45.1 (26.5)	-14.8 (-24.4, -5.2; p = 0.003)

Difference compared to placebo calculated using repeated measures ANOVA model

### Gastrointestinal symptoms

Table 5 below sets out the proportions experiencing any gastrointestinal symptoms. These were recorded as nausea, abdominal cramping, distension or flatulence. The proportions experiencing each symptom are also recorded in Table 5 for descriptive purposes. There were no statistically significant differences between any of the treatment groups in the odds of experiencing one or more gastrointestinal symptoms through repeated measures logistic regression.

**Table 5:** Side effects experienced by placebo / ME dosage.

	Proportion experiencing one or more gastrointestinal symptoms	Proportion experiencing nausea	Proportion experiencing abdominal cramping	Proportion experiencing distension	Proportion experiencing flatulence
placebo	21/37 (56.8%)	6/37 (16.2%)	7/37 (18.9%)	15/37 (40.5%)	18/37 (48.6%)
125mg	23/37 (62.2%)	8/37 (21.6%)	7/37 (18.9%)	9/37 (24.3%)	16/37 (43.2%)
250mg	20/37 (54.0%)	6/37 (16.2%)	8/37 (21.6%)	13/37 (35.1%)	17/37 (45.9%)
500mg	20/37 (54.0%)	4/37 (10.8%)	8/37 (21.6%)	12/37 (32.4%)	19/37 (51.4%)

## Discussion

In this randomised, double-blind, placebo-controlled phase 2 dose ranging trial, carried out in healthy normoglycaemic individuals, we have shown that ME can decrease total glucose and insulin rises without significant side effects. Moreover, Reducose, a proprietary mulberry leaf extract demonstrates a classical dose response curve with significant effects over placebo. Importantly, we did not find any significant differences between the treatment groups in the odds of experiencing one or more gastrointestinal symptoms. We did not observe an increased incidence of gastrointestinal side effects from ME with increasing dose and no subjects dropped out of the study due to side effects. Furthermore, a previous study using ME three times daily for twelve weeks also reported no adverse events [27].

In a crossover trial it is important to ensure that there was no carry over effects. In addition to animal data on the short half-life of DNJ of approximately two hours [25], we performed analysis using the trial data. We calculated carry-over effects using the omnibus test (a measure reflecting the degree to which the study design allows the treatment effects to be estimated independently of the carryover effects) and we found no evidence of a carryover effect in the trial (F = 1.04, p = 0.377). We also tested for a treatment by period interaction and the terms were not significant. However, the trial may not have been powered to detect carry-over effects.

A particular finding from this study was that the ME did not appear to affect the average glucose or insulin responses until 30 minutes after ingestion. Other studies using ME have shown a reduction in glucose and insulin responses occurring more rapidly after ingestion when ME was not encapsulated [22]. The capsule material used in this study was hydroxypropyl methylcellulose (HPMC) and in vitro studies have shown that this capsule material can impact (and significantly lengthen the) disintegration and dissolution behaviour of plant extracts [28]. It is possible that the choice of capsule material led to a delay in the release of the active contents and a reduction in effect size.

Mulberry leaf extracts (ME) have a long history of safe and side-effect free use. It is thought that iminosugars such as 1-deoxynojirimycin (DNJ), a reversible, competitive natural *α*-glucosidase inhibitor, are the main active components [10] and therefore ME may have a similar mode of action to acarbose [29]. Acarbose can be an adjunct to diet and exercise as monotherapy when other oral antidiabetic agents are contraindicated, or in any combination of oral antidiabetic drugs and insulin in the management of type 2 diabetes mellitus. Acarbose has been shown to reduce HbA_1C_ and the results of several large trials evaluating cardiovascular outcomes are awaited [30]. Gastrointestinal side effects are the main limiting factor in the clinical use of acarbose, leading to high rates of non-compliance and discontinuation [30]. Gastro-intestinal side effects are also common and can be problematic occurrences with other antidiabetic agents such as metformin [31].

Previous research has demonstrated that Mulberry leaf extracts (ME) can reduce postprandial glucose and insulin levels [16] but the clinical interpretation of many trials have been limited by poor study design and small numbers of subjects. In addition to the proposed direct effect of ME on *α*-glucosidase (amongst other enzymes) and on sugar and carbohydrate absorption, the ability of ME to reduce insulin rises is important in that whole-body glucose uptake progressively increases with higher rates of systemic insulin concentrations [32,33]. Indeed, suppression of insulin secretion (without dietary or exercise intervention) may lead to loss of body weight and fat mass [34]. Long-term administration of ME has produced a dose-dependent decrease in body weight and hepatic lipid accumulation in mice [19].

ME contains several herbal glycoproteins and in addition to *α*-glucosidase inhibition, in vitro studies have demonstrated the presence of fagomine in ME which may be responsible for enhanced insulin sensitivity to glucose metabolism [23]. ME has also been shown to produce hypolipidemic effects in patients with diabetes [21]. Interestingly, α-glucosidase inhibitors augment incretin hormone secretion and thus, enhanced β-cell function could, in part, explain these beneficial effects on glucose homeostasis. By altering gut microbiota flora, α-glucosidase inhibitors could also exert beneficial effects on glucose tolerance [35].

The enzyme binding kinetics of ME require further elucidation in relation to its potential pragmatic efficacy including its activity during the consumption of complex carbohydrates along with fats, which may delay gastric emptying, as may varied eating patterns such as snacking. Long-term trials are needed to investigate the safety and impact of ME on long-term glucose tolerance. Glucose-lowering agents show ethnic variations and future work should include assessment in more ethnically diverse populations.

### Limitations

We only evaluated the short-term effects of ME using single doses and longer administration and follow-up periods would be required to determine if there is a sustained effect or other potential side effects. We also used a test carbohydrate in fasting individuals and did not evaluate the pragmatic effects of ME with carbohydrates mixed with fats and proteins. The subjects in the study were not on medications which may impact on the efficacy of ME such as proton pump inhibitors or other agents disrupting stomach pH or gastric emptying. Although the use of capillary blood glucose has been validated and is recommended for determining glycaemic responses (ISO 26642: 2010(E)), there is less evidence for the robustness of capillary insulin. We did however observe a high degree of correlation between respective glucose and insulin responses suggesting that capillary insulin could be a valid measure. Although we have demonstrated that ME can reduce glucose and insulin rises in healthy volunteers with non-impaired glucose homeostasis, the results should be interpreted with caution regarding dysglycaemia.

## Conclusion

We have demonstrated that ME substantially reduces the increase in plasma glucose after ingestion of maltodextrin over 120 minutes. The pattern of effect demonstrates a classical dose response curve with significant effects over placebo. Importantly, total insulin rises were also significantly suppressed over the same period. There were no statistically significant differences between any of the treatment groups in the odds of experiencing one or more gastrointestinal symptoms indicating that ME is well tolerated. Mulberry extract may have multiple modes of action and further studies are necessary to evaluate the potential of ME for the prevention of type 2 diabetes and regulation of dysglycaemia.

## Supporting information

S1 Checklist (DOC)

S1 Protocol (PDF)

S1 Dataset (XLSX)
