# Supplementary material for: Mulberry-extract improves glucose tolerance and decreases insulin concentrations in normoglycaemic adults: Results of a randomised double-blind placebo-controlled study
Source: PLoS One. 2017 Feb 22;12(2):e0172239. doi: 10.1371/journal.pone.0172239 (PMC5321430; doi:10.1371/journal.pone.0172239)
Supplement: S1 Protocol — (PDF) [file pone.0172239.s002.pdf]

Mulberry-extract to mod**UL**ate **B**lood glucos**E** **R**esponses in no**R**mogl**Y**caemic adults **(MULBERRY)**: study protocol for a randomized controlled trial

Mark Lown[1] (corresponding author) (m.lown@soton.ac.uk) , Richard Fuller[1] (r.fuller@soton.ac.uk) , Helen Lightowler[2] (hlightowler@brookes.ac.uk) , Ann Fraser[2] (afraser@brookes.ac.uk) , Andrew Gallagher[3] (agallagher@phynova.com) , Beth Stuart[1] (bls1@soton.ac.uk) , Christopher D Byrne[4] (c.d.byrne@soton.ac.uk) , George Lewith[1] (gl3@soton.ac.uk)

[1] Primary Care & Population Sciences, Faculty of Medicine, University of Southampton, Aldermoor Health Centre Southampton SO16 5ST

[2] Functional Food Centre, Oxford Brooks University, Gipsy Lane Campus, Headington, Oxford, OX3 0BP

[3] Chief Operating Officer, Phynova Group Ltd, 16 Fenlock Court, Blenheim Office Park, Long Hanborough, OX29 8LN

[4] Professor of Endocrinology and Metabolism. University of Southampton and University Hospitals Southampton, UK

Abstract

Background: Worldwide sugar consumption has tripled during the last fifty years. High sugar intake is associated with weight gain, increased incidence of diabetes and has been linked with increased cardiovascular mortality. Reducing the health impact of dietary sugar and poor quality carbohydrate intake is a public health priority. IminoNorm®, a proprietary mulberry leaf extract (ME) may reduce blood glucose responses following dietary sugar and carbohydrate intake by reducing absorption of glucose from the gut. Previous research has shown that ME can reduce blood glucose and improve insulin responses in healthy subjects and also in subjects with raised fasting blood glucose levels. Mulberry leaf has an excellent safety profile. This pilot study will test a novel safe water soluble product in normoglycaemic adults in the UK to determine if it can reduce glucose absorption without increasing plasma insulin concentration.

Methods/Design: The trial will be a double blind, individually randomised, four-arm single dose crossover design to test the effect of three doses of ME in order to determine efficacy, dose response relationship and gastrointestinal side effects with respect to placebo. A total of 40 subjects will participate in this study and attend for four visits receiving each of the four interventions in random order.

Discussion: We aim to test the evidence that mulberry leaf extract can reduce blood glucose without a disproportionate increase in blood insulin responses in healthy individuals in a high-quality research study based in the UK. It is hoped that this will lead to further randomised controlled trials and an effective dietary supplement to lower blood glucose concentrations.

Trial Registration: ISRCTN: ISRCTN 14597438 (21/04/2015)

Keywords: Mulberry, Glycaemic Response, Insulinaemic Response

Background

Excess calorie intake including those from sugar can make a significant contribution to becoming overweight [1, 2] and thus increase the risk of developing Type 2 diabetes mellitus (T2DM) [3, 4]. In 2013 a large long-term European study investigating the effect of diet on health [5] found an association between the amount of sugary soft drinks people had and their risk of Type 2 diabetes. In this study weight gain had a large effect on diabetes risk and sugary drinks had a small effect on diabetes risk even after Body Mass Index (BMI) was corrected for. The global rise in T2DM is linked to the metabolic syndrome (dyslipidemia, hypertension, insulin resistance), and obesity is thought to be one of the greatest risk factors for metabolic syndrome and T2DM [6]. Dietary sugars and carbohydrates play a significant role as calories from these foods promote fat storage and hunger [7]. A recently completed review of nutrition and its impact on T2DM

concluded that dietary restriction of carbohydrate intake is the single most effective approach to manage T2DM [8]. It is estimated that more than 1 in 17 people in the UK have diabetes (diagnosed or undiagnosed) [9] and thus reducing the health impact of dietary sugar and poor quality carbohydrate intake is a public health priority. Herbal agents could be quite effective in the management of carbohydrates in the normal diet and in reducing post-prandial blood glucose [11].

Mulberry (*Morus alba*) leaves have been used in traditional Chinese medicine (TCM) for several millennia and its use was first recorded in around 500AD in the *Divine Husbandman's Classic of the Materia Medica* [10]. It has also been used for more than 750 years in Japan as an infusion tea [11]. It is used by some human communities for food purposes and in several Asian countries as a herbal tea. Reports have shown that the leaves are nutritious and non-toxic [12]. The Chinese Ministry of Health and the Taiwanese Bureau of Food Safety recognise *Morus alba* leaves as both a food and a medicine [13]. Mulberry leaf extracts (ME) have a long history of safe use globally for normalizing post-prandial blood glucose, and it is thought that iminosugars such as 1-deoxynojirimycin (DNJ), a reversible, competitive natural α-glucosidase inhibitor, are the main active components responsible for the activities [11].

ME can significantly reduce the peak blood glucose levels and the insulin response levels (Kimura, *et al.*, 2007; Mudra *et al.*, 2007), providing protection to blood glucose metabolic function of healthy and hyperglycemic subjects [14, 15]. ME can reduce oxidative stress, glucose fluctuations during postprandial periods and more generally, glucose swings triggering effects on oxidative stress. By reducing oxidative stress, ME has the potential to slow down the process of prediabetes to diabetes and diabetes to developing complications [16, 17]. Long-term administration of ME produced a dose-dependent decrease in body weight and hepatic lipid accumulation [17], stimulated skeletal muscle 5'-AMP-activated protein kinase activity acutely without changing the intracellular energy status [18], suppressed the elevation of postprandial blood glucose and cholesterol in humans [14] and exhibited potential hypoglycemic and hypolipidemic effects in diabetic patients [19]. Therefore, ME may help reduce blood glucose peaks caused by the ingestion sugary or carbohydrate rich foods and drinks. Mulberry tea has been shown to suppress the postprandial rise of blood glucose levels after 90 minutes of its consumption in T2DM subjects [20].

The aim of this study is to determine the effect of three doses of a proprietary water extract of mulberry leaves standardized to contain 5% DNJ (IminoNorm®) versus placebo on blood glucose and insulin responses when co-administered with 50g maltodextrin in normoglycaemic healthy adults. We also aim to determine the gastrointestinal tolerability of the mulberry extract using both normal (250mg IminoNorm® containing 12.5mg DNJ), half (125mg IminoNorm® containing 6.75mg DNJ), and double (500mg IminoNorm® containing 25mg DNJ) the normal dose. Maltodextrin is a dietary starch with a high glycaemic index, is commonly added to many foods and beverages and has been used as a test carbohydrate in similar studies [21]. We hypothesise that ME will reduce glucose excursions without causing a disproportionate increase in insulin release and thus its ingestion may confer protection against the development of diabetes, although further trials would be required to determine its long-term efficacy.

Methods / Design

Objectives

The primary objective is to determine the effect of three doses of mulberry-extract (ImінoNorm®) versus placebo on blood glucose and insulin responses when co-administered with 50g maltodextrin in normoglycaemic healthy adults. We also aim to determine the gastrointestinal tolerability of the mulberry extract using both normal (250mg IminoNorm® capsule containing 12.5mg 1-deoxynojirimycin), half (125mg IminoNorm® containing 6.75mg DNJ), and double (500mg IminoNorm® containing 25mg DNJ) the normal dose. The ME (IminoNorm®) is provided by Phynova Group Ltd, UK.

Hypothesis

Our primary hypothesis is that an appropriate dose of mulberry extract co-administered with oral maltodextrin will reduce the incremental area under the curve for plasma glucose concentration over 120 minutes in normoglycaemic adults in a dose-dependent manner when compared to co-administration with placebo. Our secondary hypothesis is that mulberry extract co-administered with oral maltodextrin will not disproportionately increase the incremental area under the curve for plasma insulin concentration over 120 minutes in normoglycaemic adults compared to co-administration with placebo.

Study Design

A double-blind, randomised, repeat measure, crossover design trial will be used to study the glycaemic response (GR) and insulinaemic response (IR) to three products: one reference product and three test products. Participants will act as their own controls, i.e. each participant will test all four products. The trial will be conducted by the Functional Food Centre at Oxford Brookes University. Ethical approval for the study has been obtained from the University Research Ethics Committee (UREC) of Oxford Brookes University, Faculty of Health and Life Sciences, Headington Campus, Gipsy Lane, Oxford, OX3 0BP, UK. Participants will be given full details of the study protocol and the opportunity to ask questions. All participants will give written informed consent prior to participation.

Participants

A sample size of 40 participants will be recruited to allow for any dropouts during the study. Healthy male and female adult participants (aged 18-60 years) will be recruited for the study in order to determine if ME can have an affect on subjects without impaired glucose tolerance.

Exclusion Criteria

The exclusion criteria of the MULBERRY trial are listed in table 1.

In addition, participants will be excluded if they are unable to comply with experimental procedures or do not follow GR and IR testing safety guidelines.

Suspension Criteria

All adverse events will be monitored and recorded from the time of the screening visit through 24 hours after treatment visit. Subjects may be excluded or have the study product discontinued if the investigator decides discontinuation of the study product is in the best medical interest of the subject (e.g., due to noncompliance, changes in medications or dietary supplements, an adverse event or serious adverse event, or a change in medical status) or if the subject requests withdrawal from the study.

Sample Size

A recent study in India (which is yet to be published) in 12 healthy individuals age 18-25 using 250mg ME dose showed a reduction in the glycaemic index of maltodextrin by 58% when compared to placebo. A sample size of 30 would allow over 90% power to detect a difference of a similar size. Being more conservative and allowing for a smaller difference to be detected in the lower concentration doses, 30 participants would still allow at least 80% power to detect a difference of 25% in the incremental Area Under the Curve (iAUC). In order to account for a potential loss to follow up, and the possibility that our sample size may be inaccurate as it is based on a small pilot sample we will therefore recruit 40 participants.

Randomisation

Researchers recruiting the participants will be unaware of the allocation sequence (concealed allocation). Assignment of participants to a participant number will be done according to their chronological order of enrolment in the study. The allocated participant number will identify the participants and their corresponding intervention sequence. A code break for the randomisation will be kept by the Principal Investigator and will only be broken in terms of a serious adverse event.

Blinding (Treatment / Placebo)

Four products will be tested in this study - one placebo reference product (four capsules containing 125mg microcrystalline cellulose) and three test products containing different doses of mulberry extract (test product groups receive either 1, 2, or 4 capsules containing 125mg ME, with either 3, 2, or 0 placebo capsules respectively so that participants always take 4 capsules). The study products are double blinded to both investigator and subject. Each test/reference product will be co-administered with 50g maltodextrin dissolved in 250ml water. Products will be produced by Phynova and delivered to the study centre labelled as 'Product variant A', 'Product variant B', 'Product variant C' and 'Product variant D' with sealed envelopes labelled with participant number and the intervention sequence for the respective participant.

Study Period

In each participant, the reference product and test products will be tested in random order on (four) separate days, with at least a two-day gap between measurements to minimise carry over effects. The study schedule is summarised in Table 2.

Outcomes

Primary outcome:

iAUC for plasma glucose concentration over 120 minutes.

Secondary outcomes:

- iAUC for plasma insulin concentration over 120 minutes.
- Gastrointestinal tolerability – abdominal bloating may be experienced due to reduced carbohydrate absorption. Gastrointestinal symptoms will be measured via questionnaire for 24 hours following each study visit. Subjects will use a 5-point scale to rate stool consistency for each bowel movement for 0-24 h after the study product consumption. The five-point scale includes: 1=watery, 2=loose/mushy, 3=soft, 4=formed, 5=hard. Frequency and intensity will be set to a 10-centimeter (cm) line scale (0 representing "Absent" for frequency and "Usual" for intensity; 10 representing "More than usual" for frequency and "Severe" for intensity).

Recruitment

Participants will be recruited following local advertisements and all participants will be given full details of the study protocol and the opportunity to ask questions. All participants will give written informed consent prior to participation and will be paid £10 per visit, on completion of all four visits. This has been determined as an appropriate amount to cover and travel costs and the time spent during each visit.

Procedures / Study Schedule

The study will be conducted by Good Clinical Practice (GCP) certified researchers. The reference product and test products will be administered to participants in a randomised, repeated measures design. On the day prior to a test, participants will be asked to restrict their intake of alcohol and caffeine-containing drinks and to restrict their participation in intense physical activity (for example, long periods at the gym, excessive swimming, running, aerobics). Participants will also be told not to eat or drink after 10.00 pm the night before a test, although water will be allowed in moderation. Participants will be studied in the morning after an overnight fast. Participants will consume the products at a comfortable pace, within 5 minutes. The reference product and test products will be served with 50g maltodextrin dissolved in 250 ml water. Participants will remain sedentary during each test session and will not consume any additional food or fluid. Participants will be instructed to record stool consistency for the first bowel movement after their visit and the frequency and intensity of gastro intestinal symptoms for 0-24 hours after the study product consumption.

All anthropometric measures will be taken, using standard methods, in the morning, after an overnight fast.

- Height and weight
- BMI will be calculated using the standard formula: weight (kg) / height (m^2)
- Waist and hip circumference

The glycaemic response method used is adapted from that described by Brouns *et al*. [22] and is carried out in accordance with the ISO 26642:2010 standards. Blood measurements will be taken at -5 min and 0 min before consumption of the reference product/test products and the baseline value taken as a mean of these two values. Further blood measurements will be taken at 15, 30, 45, 60, 90 and 120 minutes after starting to eat. Blood glucose will be measured using the HemoCue Glucose 201+ analyser (HemoCue® Ltd). The same time points will be used for determining insulin levels. At each test time point, 300 μL of capillary blood (from finger pricks) will be obtained. Finger pricks will be made using the Unistik 3 single-use lancing device (Owen Mumford, Woodstock, UK) and blood will be collected into chilled Microvette® capillary blood collection tubes treated with di Potassium EDTA (CB 300 K2E; Sarstedt Ltd., Leicester, UK). The Microvette® tubes will then be centrifuged and 200 μL of the supernatant plasma obtained. Insulin concentrations in the plasma samples will be determined by electrochemiluminescence immunoassay using an automated analyzer (Cobas® E411; Roche diagnostics, Burgess Hill, UK). The Cobas® system is a reliable method of plasma insulin determination. The unit of measurement is μU/ml. Sufficient blood is taken to enable 2 sets of analysis to be performed at every time point thus greatly reducing the likelihood of missing data.

Statistical Analysis

This is the first rigorous study of IM and should therefore be considered as a pilot study from a statistical perspective. We will calculate the incremental area under the curve for all 4 study products and compare using repeated measures ANOVA to determine whether there is a statistically significant difference in plasma glucose levels and in secondary outcome measure over the 120 minute period. All models will control for the possible confounding effects of sex and BMI. The presence/absence of gastrointestinal symptoms in the 24 hours following the study visit will be assessed using logistic regression models. We anticipate a low likelihood of missing data (as sufficient blood is taken for a second analysis to be performed if the first analysis fails) and our glucose values will be measured in real time. We anticipate monophasic or biphasic glucose and insulin curve responses and will use a 2nd order interpolation method for missing data, provided that no other data is missing from the two neighbouring time points at either side of a missing data set and that the baseline and end data values are obtained. If the baseline or end points are missing or more than one data point within five neighbouring time points, this data set will be deemed unsuitable and not be included in the analysis.

Discussion

In this paper, we have suggested the clinical trial design of a dietary supplement to reduce glucose excursions without a disproportionate increase in insulin release. This pilot study will influence further work on a larger scale RCT. In this study we aim to confirm the standard dose of IminoNorm that can be consumed with food. In addition, we aim to determine the magnitude of clinical effect and to measure the frequency and intensity of any gastrointestinal side effects. Based on the results, we will aim to design a larger RCT to investigate the protective effect of ME on the development of pre-diabetes and diabetes in at risk individuals or the population as a whole. The treatment effect size may be used to determine the sample size of a larger study based on similar studies on the prevention of T2DM As mentioned previously, ME may also have a positive effect on lipid profiles and we may decide to include further endpoints in future larger trials. Glucose-lowering agents show ethnic variations and future work should include assessment in more ethnically diverse populations.

Trial Status

We have now finished recruiting and most of the participants have completed all 4 visits.

List of Abbreviations

ME: Mulberry Extract, T2DM: Type 2 Diabetes Mellitus, BMI: Body Mass Index, TCM: Tradition Chinese Medicine, DNJ: 1-deoxynojirimycin, GR: Glycaemic Response, IR: Insulinaemic Response, UREC: University Research Ethics Committee, iAUC: Integral Area Under the Curve, GCP: Good Clinical Practice, ANOVA: Analysis of Variance, RCT: Randomised Controlled Trial.

Competing Interests

The authors are funded by a Technology Strategy Board / Innovate UK grant to undertake this work with Phynova (as the applicant) and our role is solely that of researchers

Author's Contributions

RF, AG and GL participated in the initial design of the study. BS participated in the statistical analysis. CB is the chief investigator and contributed to the study design. HL and AF participated in the final review of the protocol, the recruitment of participants and the running of the trial at Oxford Brookes. ML contributed to the protocol and drafted the manuscript. All authors read and approved the final version of this manuscript.

Acknowledgements

The work has been funded by a Technology Strategy Board/Innovate UK grant.

References

[1] Te Morenga L, Mallard M, Mann J. Dietary sugars and body weight: systematic review and meta-analyses of randomised controlled trials and cohort studies. BMJ. 2012 Jan 15;346:e7492. doi:10.1136/bmj.e7492.

[2] National Institute for Health and Care Excellence. NICE Guideline CG43. Obesity: guidance on the prevention, identification, assessment and management of overweight and obesity in adults and children. December 2006. https://www.nice.org.uk/guidance/cg43. Accessed 15 July 2015.

[3] National Institute for Health and Care Excellence. NICE Guideline PH35. Preventing Type 2 diabetes: population and community- level interventions. May 2011. http://www.nice.org.uk/guidance/ph35. Accessed 15 July 2015.

[4] National Institute for Health and Care Excellence. NICE Guideline PH 38. Preventing Type 2 diabetes: risk identification and interventions for high risk individuals. July 2012. http://www.nice.org.uk/guidance/ph38. Accessed 15 July 2015.

[5] InterAct Consortium, Romaguera D, Norat T, Wark PA, Vergnaud AC, Schulze MB, et al. Consumption of sweet beverages and type 2 diabetes incidence in European adults: results from EPIC-InterAct. Diabetologia. 2013 Jul;56(7):1520-30. doi:10.1007/s00125-013-2899-8.

[6] Basu S, Yoffe P, Hills N, Lustig RH. The Relationship of Sugar to Population-Level Diabetes Prevalence: An Econometric Analysis of Repeated Cross-Sectional Data. PLoS ONE 8(2): e57873. doi:10.1371/journal.pone.0057873

[7] Malhotra A, Noakes T, Phinney S. Br J Sports Med. 2015 Apr 22. pii: bjsports-2015-094911. doi:10.1136/bjsports-2015-094911.

[8] Feinman RD, Pogozelski WK, Astrup A, Bernstein RK, Fine EJ, Westman EC, et al. Dietary carbohydrate restriction as the first approach in diabetes management : Critical review and evidence base. Nutrition. 2015 Jan;31(1):1-13. doi:10.1016/j.nut.2014.06.011.

[9] Quality and outcomes framework (QOF) 2012/3

England http://www.hscic.gov.uk/article/2021/Websitesearch?q=quality+and+outcomes+framewo rk&go=Go&area=both

England: http://www.hscic.gov.uk/qof

Wales http://wales.gov.uk/topics/statistics/headlines/health2013/general-medical-services- contract-quality-outcomes-framework-2012-13/?lang=en

Wales: http://gov.wales/statistics-and-research/general-medical-services-contract/?lang=en

Scotland http://www.isdscotland.org/Health-Topics/General-Practice/Quality-And-Outcomes- Framework

Scotland: http://www.isdscotland.org/Health-Topics/General-Practice/Quality-And-Outcomes-Framework/

Northern Ireland http://www.dhsspsni.gov.uk/index/stats_research/stats-resource/stats-gp- allocation/gp_contract_qof.htm

[10] Bensky D, Gamble A. Chinese Herbal Medicine: Materia Medica, revised edition. Seattle: Eastland Press; 1993.

[11] Oku T, Yamada M, Nakamura M, Sadamori N, Nakamura S. Inhibitory effects of extractives from leaves of Morus alba on human and rat small intestinal disaccharidase activity. Br J Nutr. 2006 May;95(5):933-8.

[12] Srivastava S, Kapoor R, Thathola A, Srivastava RP. Mulberry (Morus alba) leaves as human food: a new dimension of sericulture. Int J Food Sci Nutr. 2003 Nov;54(6):411-6.

[13] Chau CF, Wu SH. The development of regulations of Chinese herbal medicines for both medicinal and food uses. Trends Food Sci. Technol. 2006;17:313-323.

[14] Kimura T, Nakagawa K, Kubota H, Kojima Y, Goto Y, Yamagishi K, et al. Food-grade mulberry powder enriched with 1-deoxynojirimycin suppresses the elevation of postprandial blood glucose in humans. J Agric Food Chem. 2007 Jul 11;55(14):5869-74.

[15] Monnier L, Mas E, Ginet C, Michael F, Villon L, Cristol JP, et al. Activation of oxidative stress by acute glucose fluctuations compared with sustained chronic hyperglycemia in patients with type 2 diabetes. JAMA. 2006 Apr 12;295(14):1681-7.

[16] Kawamori R, Tajima N, Iwamoto Y, Kashiwagi A, Shimamoto K, Kaku K, et al. Voglibose for prevention of type 2 diabetes mellitus: a randomised, double-blind trial in Japanese individuals with impaired glucose tolerance. Lancet. 2009 May 9;373(9675):1607-14. doi:10.1016/S0140-6736(09)60222-1.

[17] Wu T, Qi X, Liu Y, Guo J, Zhu R, Chen W, et al. Dietary supplementation with purified mulberry (Morus australis Poir) anthocyanins suppresses body weight gain in high-fat diet fed C57BL/6 mice. Food Chem. 2013 Nov 1;141(1):482-7. doi:10.1016/j.foodchem.2013.03.046.

[18] Ma X, Iwanaka N, Masuda S, Karaike K, Egawa T, Hamada T, et al. Morus alba leaf extract stimulates 5'-AMP-activated protein kinase in isolated rat skeletal muscle. J Ethnopharmacol. 2009 Feb 25;122(1):54-9. doi:10.1016/j.jep.2008.11.022.

[19] Andallu B, Suryakantham V, Lakshmi B, Reddy GK. Effect of mulberry (Morus indica L.) therapy on plasma and erythrocyte membrane lipids in patients with type 2 diabetes. Clin Chim Acta. 2001 Dec;314(1-2):47-53.

[20] Banu S, Jabir NR, Manjunath NC, Khan MS, Ashraf GM, Kamal MA, et al. Reduction of post-prandial hyperglycemia by mulberry tea in type-2 diabetes patients. Saudi J Biol Sci. 2015 Jan;22(1):32-6. doi:10.1016/j.sjbs.2014.04.005.

[21] Mudra M, Ercan-Fang N, Zhong L, Furne J, Levitt M. Influence of mulberry leaf extract on the blood glucose and breath hydrogen response to ingestion of 75 g sucrose by type 2 diabetic and control subjects. Diabetes Care. 2007 May;30(5):1272-4.

[22] Brouns F, Bjorck I, Frayn KN, Gibbs AL, Lang V, Slama G, et al. Glycaemic index methodology. Nutr Res Rev. 2005 Jun;18(1):145-71. doi:10.1079/NRR2005100.

Additional File

An additional file has been included which details recommended items to address in a clinical trial protocol and related documents.

Table 1 Exclusion criteria of the Mulberry trial

Exclusion Criteria
1. Aged < 18 or > 60 years
2. Pregnant or lactating
3. Body mass index (BMI) < 20kg/m^2 and > 30kg/m^2
4. Fasting blood glucose value > 6.1 mmol/l
5. Any known food allergy or intolerance including mulberry extract
6. Medical condition(s) or medication(s) known to affect glucose regulation or appetite and/or influence digestion and absorption of nutrients
7. Known history of diabetes mellitus (Type I/II) or the use of antihyperglycaemic drugs or insulin to treat diabetes and related conditions
8. Use steroids, protease inhibitors or antipsychotics (all of which have major effects on glucose metabolism and body fat distribution)
9. Current oral hypoglycaemic use
10. Symptomatic IBS
11. History of renal or liver diseases
12. History of clotting or bleeding disorders
13. Taken antibiotics in last 3 weeks prior to screening
14. Taking daily medications or dietary supplements that are not suitable for the study in the opinion of the PI
15. Anaemia
16. Subject to a major medical or surgical event requiring hospitalization within the preceding 3 months
17. Current participation in another clinical study.

Table 2 Summary of MULBERRY trial schedule

	Screening Visit	Visit 1	Visit 2	Visit 3	Visit 4
Informed Consent	•				
Demographic Info (age, contact details etc)	•				
Medical History	•				
Inclusion/Exclusion Criteria	•				
Compliance Check (fasting, drugs etc)		•	•	•	•
Physical Exam (BMI, waist circ. etc)		•	•	•	•
Consume product / placebo + drink		•	•	•	•
Finger-prick glucose & insulin testing		•	•	•	•
GI Questionnaire		•	•	•	•
Adverse Event Monitoring		•	•	•	•
